# What is the future of acromegaly therapy?

**DOI:** 10.1210/clinem/dgag144

**Published:** 2026-04-08

**Authors:** Katharina Schilbach, Christian J Strasburger

**Affiliations:** Medizinische Klinik und Poliklinik IV, LMU Klinikum, Munich 80336, Germany; Deggendorf Institute of Technology, Faculty of Applied Healthcare Sciences, Deggendorf 94469, Germany; Department of Endocrinology and Metabolism, Charité—Universitätsmedizin Berlin, corporate member of Freie Universität Berlin, Humboldt-Universität zu Berlin, Berlin Institute of Health, Berlin 10117, Germany

**Keywords:** acromegaly novel therapy, Debio 4126, ALXN2420, MAR002, CAM2029

## Abstract

Acromegaly is a chronic multisystem disorder in which biochemical control remains suboptimal for many patients. Even when control is achieved, treatment burden is often high despite surgery and a spectrum of established safe and effective therapies. In this narrative review of multinational late-stage clinical trials through October 2025, we summarize drug developments that may broaden individualized medical strategies. Oral somatostatin receptor ligands are now available in the United States, underscoring a shift toward more convenient long-term management. In parallel, non-oral innovations continue to advance. Octreotide subcutaneous depot (CAM2029) maintains biochemical control with acceptable safety and may reduce treatment burden. Emerging pituitary-directed therapy with longer application intervals (Debio 4126) also shows pharmacologic activity consistent with effective disease control. Growth hormone (GH) receptor–directed agents (ALXN2420, MAR002) likewise demonstrate activity aligned with considerable biochemical and clinical benefit. Collectively, these agents may address unmet needs by broadening the scope of options to control production of hepatic insulin-like growth factor I (IGF-I) and to attenuate additional extrahepatic GH signaling. They may also help ease the practical burden of chronic therapy.

Acromegaly is a chronic, multisystem disorder in which persistent excess of growth hormone (GH) and insulin-like growth factor I (IGF-I) drives morbidity and mortality. Current management targets include normalization of age-adjusted IGF-I levels in circulation, somatotroph adenoma control, and resolution of individual disease-specific symptoms and comorbidities. Updated consensus recommendations emphasize a multidisciplinary approach and shared decision-making that incorporates comorbidities and patient-reported outcomes (1).

Despite these advances, persistent unmet needs include broader access to effective noninjectable or other less invasive therapies that avoid strict food-related dosing requirements (2-4); persistent discordance between biochemical control and patient-reported outcomes (2, 5); prolonged diagnostic delay with consequent morbidity and mortality (6); limited access in some regions to high-volume pituitary surgery within multidisciplinary centers (7, 8); and substantial geographic variability in medication availability and reimbursement (9). The somatostatin receptor ligands (SRLs), octreotide and lanreotide, cornerstones of medical therapy, are associated with variable biochemical and adenoma responses and have a nontrivial treatment burden (10). According to the latest consensus recommendations, pegvisomant monotherapy may be considered as a first-line treatment for selected patients, particularly those with significantly impaired glucose metabolism and no immediate concern regarding adenoma size, and especially when non- or partial responsiveness to SRLs can be anticipated (1). Non-responsiveness to SRLs can be anticipated from histopathologic and imaging markers such as a sparsely granulated somatotroph adenoma, low somatostatin receptor 2 (SST2) immunostaining, and T2-weighted magnetic resonance imaging (MRI) hyperintensity, as well as from very high baseline IGF-I/GH concentrations, although the predictive value of these features is not uniformly reliable (11). When the likelihood of nonresponse is high, first-line pegvisomant may be considered within an individualized, shared decision-making framework (12).

Pasireotide is a second-line therapeutic option for patients who remain biochemically and/or clinically inadequately controlled on first-line medical therapy, particularly when adenoma shrinkage/control is a priority and characteristics predicting poor response to octreotide/lanreotide are present (1).

## Recently approved drugs

New SRLs have the potential to recalibrate the equilibrium among efficacy, safety, and treatment burden. Oral choices include capsules (Mycapssa®) and paltusotine tablets (Palsonify^™^), which is US Food and Drug Administration (FDA)-approved (September 2025) but not yet European Medicines Agency (EMA)-approved (13-16).

Efficacy and safety of paltusotine as an oral once-daily non-peptidergic medication were demonstrated in 2 placebo-controlled phase 3 studies investigating patients with controlled (IGF-I ≤ 1.0× upper limit of normal [ULN]) (17) and uncontrolled (IGF-I ≥ 1.3 × ULN) (18) acromegaly disease activity. The 2 studies are reviewed in more detail in this supplement by A. Ioachimescu and F. Castinetti.

A ready-to-use octreotide subcutaneous (SC) depot using FluidCrystal^®^ technology (CAM2029, Oczyesa^®^) approved by the EMA (June 2025) was introduced in Europe (Camurus, Lund, Sweden, October 2025) (19). In ACROINNOVA-1, a 24-week, multinational, randomized, double-blind, placebo-controlled phase 3 trial, 72 adults with acromegaly who were biochemically controlled at screening on stable octreotide or lanreotide (IGF-I≤ ULN) were randomized 2:1 to monthly CAM2029 (octreotide SC depot, 20 mg) or placebo to test maintenance of biochemical control (20). The primary endpoint was the proportion of patients with IGF-I ≤ 1.0 × ULN at the end of the treatment phase. With CAM2029, 72.2% of patients reached this goal as compared with 37.5% with placebo (risk difference 34.6%; 95% confidence interval [CI] 11.3-57.9; *P* = .0018). The key secondary endpoint (IGF-I ≤ ULN at week 22/24 plus mean GH < 2.5 µg/L at week 24) also favored CAM2029 (70.0% vs 37.5%; *P* = .0035). Average IGF-I levels were stably controlled with CAM2029 while rising above the ULN with placebo. The time to loss of response favored CAM2029 (hazard ratio 0.10; 95% CI 0.04-0.28). Patient-reported outcomes improved with CAM2029 relative to baseline standard care. The Acromegaly Quality of Life Questionnaire (AcroQoL) total score increased (least-squares mean +4.69; 95% CI 1.51-7.86) (20, 21), indicating a better quality of life. Treatment satisfaction also improved, particularly related to convenience. On the Patient Satisfaction Scale, scores favored CAM2029 (mean 3.9; 95% CI 3.6-4.2 vs 3.4 2.9-3.8); however, formal statistical testing was not performed, so these findings should be considered descriptive. Safety was favorable and in line with expected octreotide effects. The overall incidence of adverse events was similar between the CAM2029 and placebo groups (78.7% vs 79.2%). Most events were mild, and injection-site reactions were the most frequently observed (20). Four patients receiving CAM2029 discontinued the drug because of adverse events. No serious adverse events were attributed to CAM2029 (20).

The open-label ACROINNOVA-2 phase 3 study evaluates CAM2029 in biochemically controlled patients as an open-label extension (OLE) of ACROINNOVA-1, as well as in patients with insufficiently controlled (IGF-I > ULN and ≤ 2 × ULN) acromegaly. In a prespecified interim analysis of the 52-week treatment phase, CAM2029 achieved biochemical disease control and improved patient-reported quality of life, with a favorable safety profile (22). Taking together both ACROINNOVA trials, CAM2029 maintained biochemical control more effectively than placebo, improved symptoms and quality of life, and showed tolerability comparable with currently available therapies. The reported results suggest that it is an effective and convenient long-acting octreotide option, and confirmation from longer-term, real-world data is anticipated.

## Therapeutic targets in acromegaly: adenoma-targeted vs GH receptor–directed approaches

### Hepatic vs extrahepatic acromegaly—a question of targeted control

Approximately 70% of IGF-I in the circulation stems from the liver (23), whereby circulating GH stimulates hepatocyte IGF-I synthesis, and serum IGF-I serves as a surrogate biomarker of integrated GH action and a principal mediator of anabolic and trophic effects (24). Insulin concentration in the portal vein drives hepatic GH receptor (GHR) expression (25). Octreotide and lanreotide inhibit insulin secretion, and besides directly reducing GH output, they indirectly lower hepatic sensitivity to GH and decrease IGF-I production by reducing portal insulin levels (26).

GH, however, also exerts direct action in multiple extrahepatic targets such as bone, cartilage, skeletal muscle, kidney, and adipose tissue (27-31). The recognition of a discordance between hepatic IGF-I output and peripheral GH signaling has motivated the conceptual framework of extrahepatic acromegaly, in which clinically relevant GH activity persists in extrahepatic tissues despite suppression of serum IGF-I levels (26, 32, 33). A complementary, putative predominantly “hepatic phenotype” of acromegaly disease activity has likewise been proposed, characterized by sustained hepatic IGF-I production despite attenuation of peripheral GH actions, as may occur under therapeutic regimens that differentially modulate GH receptor signaling across tissues (26, 32, 34, 35). Response-based phenotypes underscore that normalization of circulating IGF-I alone only incompletely reflects acromegaly disease activity, although IGF-I levels currently are the best biochemical indicator of such disease activity (33). This concept supports an integrated approach of combining suppression of GH/IGF-I bioactivity with clinical features, imaging, and, when feasible, tissue-specific endpoints of GH action to refine assessment and guide individualized management (36).

**Figure 1:**
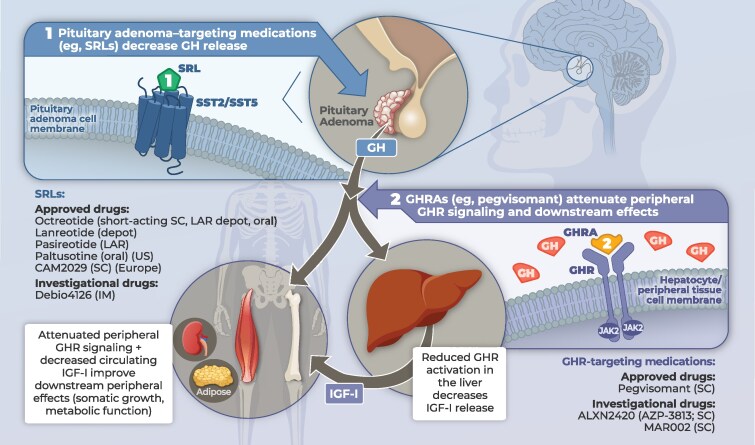
Mechanistic overview of current and emerging pharmacologic approaches for acromegaly. (1) Pituitary adenoma–targeted SRLs bind primarily SST2 and SST5 on somatotroph cells to suppress GH secretion. Drugs shown include octreotide (short-acting SC, LAR depot, oral), lanreotide (depot), pasireotide (LAR), paltusotine (oral), CAM2029 (SC), and Debio 4126 (IM). (2) GHRAs; pegvisomant and investigational agents ALXN2420 and MAR002; attenuate peripheral GHR signaling (JAK2-dependent) in liver and extrahepatic tissues, thereby reducing hepatic IGF-I output and dampening downstream GH actions (somatic growth and metabolic effects). Schematic only; not to scale. Abbreviations: GH, growth hormone; GHR, GH receptor; GHRA, GH receptor antagonist; IGF-I, insulin-like growth factor 1; IM, intramuscular; JAK2, Janus kinase 2; LAR, long-acting release; SC, subcutaneous; SRL, somatostatin receptor ligand; SST2/SST5, somatostatin receptor subtypes 2 and 5.

In distinguishing hepatic from extrahepatic components of acromegaly, somatostatin agonists primarily control the hepatic component by suppressing GH secretion, thereby reducing IGF-I production; however, an extrahepatic component may persist because GH receptors in peripheral tissues may remain excessively stimulated by circulating GH, causing symptoms and comorbidities despite seemingly acceptable biochemical control (26, 35). Therapy with a GH receptor–targeting agent, in contrast, suppresses peripheral GH signaling and thus preferentially targets extrahepatic features of the disease (37, 38). An overview of the 2 therapeutic approaches is shown in Fig. 1.

Pegvisomant, a GH receptor antagonist used to treat acromegaly (37), is a pegylated recombinant human GH analog with 9 substitutions: 8 increase affinity at GHR binding site 1, and 1 (G120K) disrupts signaling at site 2 (38, 39). Normally, GH binds a preformed GHR dimer via 2 asymmetric sites, inducing a turn of the transmembrane helices, thus separating the cytoplasmic domains, relieving Janus kinase 2 (JAK2) autoinhibition and triggering signal transducer of activator of transcription (STAT) phosphorylation (40-42). Pegvisomant binds the same extracellular epitopes but fails to induce this conformational change; intracellular GHR domains do not separate adequately, JAK2 remains inactive, and signaling is prevented (40). Thus, pegvisomant acts as a competitive GHR antagonist that prevents formation of an active GHR–GH–GHR complex and lowers circulating IGF-I levels (37, 40).

Combined SRL and GHR antagonist therapy targets both hepatic and extrahepatic components of acromegaly, thereby maximizing overall disease control. This therapeutic strategy of combination therapy has enjoyed increasing acceptance since the introduction of pegvisomant (43). Despite treatment advances, many patients with acromegaly remain either biochemically or clinically uncontrolled or face a high treatment burden, underscoring the need for future development of more effective and convenient treatment options (2).

Recent therapeutic advancements target pituitary GH-secreting adenomas through an additional octreotide formulation (Debio 4126), as well as the GHR, with 2 investigational agents (ALXN2420 and MAR002). This review focuses on these agents currently in the process of being evaluated in clinical trials. To incorporate the most recent developments in the field, we also cite conference abstracts and websites, given the limited current availability of peer-reviewed data. Table 1 summarizes drugs currently in development.

**Table 1:** Overview of newly approved products and products in development

Product	Sponsor	Active ingredient/mechanism	Route	Regulatory status	Key trial(s) (phase; NCT)	OLE phases	Current status of trials
Paltusotine (Palsonify^™^)	Crinetics	Small molecule, SST2 agonist	Oral	US FDA approved September 2025	PATHFNDR-1 (phase 3; NCT04837040); PATHFNDR-2 (phase 3; NCT05192382)	optional OLE following the double-blind core phases in both PATHFNDR-1 and PATHFNDR-2	core phases completed; OLE ongoing
CAM2029 (Oczyesa^®^)	Camurus	Octreotide SRL, SC depot using FluidCrystal^®^ technology	SC	EMA approved June 2025	ACROINNOVA-1 (phase 3; NCT04076462); ACROINNOVA-2 (phase 3; open-label, long-term safety; NCT04125836)	ACROINNOVA-2 is the open-label, long-term extension (52-week core + 52-week extension) following ACROINNOVA-1	ACROINNOVA-1 completed; ACROINNOVA-2 core completed; extension ran into 2025
Debio 4126	Debiopharm	Octreotide long-acting Q12W formulation	IM	—	OXTEND-01/Debio 4126-102 (phase 1b, open-label; NCT05364944); OXTEND-03/Debio 4126-301 (phase 3; NCT06930625)	OXTEND-03 includes an open-label Period-2 after the 36-week double-blind core	OXTEND-01 concluded, OXTEND-03 recruiting in 2025
ALXN2420 (formerly AZP-3813)	Alexion, AstraZeneca	Small, bicyclic peptide; competitive GH receptor antagonist	SC	—	ASTERIA (phase 2, randomized, double-blind, placebo-controlled, dose-range add-on to SRL; NCT07037420)	OLE to week 52 with crossover of placebo to ALXN2420 after week 15	Site start-up in 2025; status varies by registry (recruiting)
MAR002	Marea	Allosteric human monoclonal GH receptor antagonist, noncompetitive	SC	—	MAR-201 (phase 1; first-in-human SAD in healthy men; NCT07195175)	None (SAD/MAD design in healthy volunteers)	Recruiting since Aug 2025

Abbreviations: EMA, European Medicines Agency; GH, growth hormone; IM, intramuscular; MAD, multiple ascending dose; NCT, ClinicalTrials.gov identifier; OLE, open-label extension; Q12W, every 12 weeks; SAD, single ascending dose; SC, subcutaneous; SRL, somatostatin receptor ligand; SST2, somatostatin receptor 2; US FDA, United States Food and Drug Administration.

## Pituitary-directed medication in clinical trials

### Debio 4126

Debio 4126, an investigational, injectable, sustained-release formulation of octreotide enables 12-week (Q12W) intramuscular (IM) dosing, thereby reducing the treatment burden associated with monthly SRL injections (Debiopharm International SA, Lausanne, Switzerland) (44). Debio 4126 is engineered to maintain therapeutically effective octreotide exposure across an 84-day injection interval, with a concentration–time profile characterized by an initial burst, a transient nadir, and a stabilized plateau reached by approximately day 28 to support sustained somatostatin receptor (predominantly SST2 and somatostatin receptor 5 [SST5]) binding and GH suppression (45, 46). The precise composition and delivery system has not been publicly disclosed. Its design target is to achieve steady-state octreotide exposure at least comparable to standard monthly octreotide long-acting release (LAR) injections while allowing 3-month dosing (46).

The phase 1 trial presented at the 20th Congress of the European NeuroEndocrine Association 2022 (46), was an open-label, active-controlled, parallel-group, single-site study in healthy volunteers designed to characterize the pharmacokinetics (PK), pharmacodynamics (PD), safety, and tolerability of a single administration of Debio 4126 (46). Participants were assigned to 1 of 6 Debio 4126 cohorts (formulations A or B; IM or SC; 30 mg or 90 mg single dose), while a reference cohort received Sandostatin LAR^®^ 30 mg IM every 28 days for 3 doses. At the interim analysis, 90 participants were enrolled (75 received Debio 4126; 14 Sandostatin LAR^®^) and 88 completed at least 84 days (46).

Mean octreotide plasma levels were maintained ≥ 1 ng/mL throughout 12 weeks, and the concentration–time profile was broadly similar to that observed after 3 monthly injections of Sandostatin LAR^®^, with higher apparent bioavailability for Debio 4126 (46).

Dose proportionality was observed between the 30-mg and 90-mg IM cohorts;geometric-mean area under the curve (AUC^84d^) of 35.9 ng·day/mL for Debio 4126 30 mg IM, 122.5 ng·day/mL for Debio 4126 90 mg IM, and 80.4 ng·day/mL for Sandostatin LAR^®^ 3 × 30 mg (46).

Debio 4126 reduced serum IGF-I over 84 days in all cohorts and suppression after 90-mg IM was comparable with that observed with 3 separate monthly 30-mg Sandostatin LAR® injections (46).

In healthy volunteers, the safety profile of Debio 4126 aligned with class effects of approved SRLs (47). The most frequent adverse events were gastrointestinal disorders, biliary events, and injection-site reactions. No novel safety signals emerged (46).

These interim phase 1 data indicate that Debio 4126 maintains octreotide exposure at concentrations considered adequate for acromegaly control throughout a Q12W dosing interval while achieving IGF-I suppression comparable to monthly octreotide LAR, thereby supporting clinical development of a quarterly octreotide option that could reduce injection burden without compromising biochemical control. Nevertheless, interpretation is limited to the phase 1 study design (46).

OXTEND-01 (Debio 4126-102) was a phase 1b, open-label, multicenter study in patients with acromegaly in which Debio 4126 was applied at weeks 0, 12, 24, and 36 after a 4-week run-in on the prior SRL. The primary objective was descriptive PK, with IGF-I dynamics and safety as key secondary endpoints. Sixteen patients initiated treatment, and 15 completed per protocol (48). At screening, 81.3% of patients had IGF-I concentrations≤ 1.0 × ULN and 18.8% had 1.0-1.3 × ULN (48). In this open-label study with fixed-dose cohorts (30, 60, or 90 mg Q12W; 1 patient up-titrated), Debio 4126 provided sustained octreotide release over each 12-week interval (48). Octreotide exposure increased across dose groups (30→60→90 mg; no formal dose proportionality testing reported). Within a given dose, no relevant accumulation was observed over repeated dosing, and steady state was reached after the second administration (48).

Patient PK profiles resembled those previously observed in healthy volunteers. IGF-I trajectories supported biochemical maintenance on Q12W dosing when converting from monthly SRLs using the dose-matching scheme. Among evaluable participants with baseline IGF-I ≤ 1.3 × ULN, 13/14 (92.8%) remained ≤ 1.3 × ULN and those with baseline ≤ 1.0 × ULN, 9/11 (81.8%) remained≤ 1.0 × ULN at the end of the fourth interval (48). A single up-titration occurred after which escalation to 60 mg led to a modest IGF-I decrease but remained > 1.0 × ULN (48). Safety findings were consistent with somatostatin analog class effects; there were no deaths or serious adverse events (48). Treatment- related adverse events occurred in 10/16 (62.5%); events in ≥ 2 patients included injection-site reactions (erythema 12.5%, induration 12.5%, inflammation 12.5%), cholelithiasis or biliary sludge 25.0% (all asymptomatic, no interventions), and headache 12.5%. One grade 3 alanine aminotransferase (ALT) elevation resolved without treatment modification, and all other events were grade 1 or 2 (48).

In summary, the OXTEND-01 trial showed that switching patients with biochemically controlled acromegaly from monthly long-acting SRLs to Debio 4126 (administered Q12W) leads to sustained octreotide exposure with minimal accumulation and maintains IGF-I control in most evaluable participants. The tolerability profile is similar to approved current SRLs (48).

A phase 3 randomized, double-blind, placebo-controlled trial (OXTEND-03, Debio 4126-301; NCT06930625) has been recruiting since September 2025. The primary endpoint is the proportion of patients maintaining IGF-I ≤ 1.0 × ULN at week 36 during the double-blind period (Debio 4126 vs placebo).

## GH receptor antagonists

### ALXN2420

ALXN2420 (Alexion, AstraZeneca Rare Disease), an investigational GHR antagonist developed for the treatment of acromegaly, was presented as AZP-3813 in 2022 by Amolyt Pharma (49). Its current clinical development plan is focused as an add-on therapy to long-acting SRL monotherapy in adults with incomplete biochemical control (defined as IGF-I > 1.0 × ULN) (50). This molecule is a synthetic, bicyclic peptide (16 amino acids) designed to antagonize the human GHR (50). Its compact, bicyclic topology confers conformational constraint to enhance receptor binding and pharmacologic stability without requiring pegylation. ALXN2420 competitively antagonizes the GHR by blocking GH engagement and receptor activation, thus preventing downstream JAK2–signal transducer and activator of transcription 5 (STAT5) signal transduction in target tissues with subsequent reduction of IGF-I synthesis. This compound is being developed as a once daily, SC injection.

#### In vitro data

Using surface plasmon resonance (SPR) with recombinant GHR ectodomains, ALXN2420 displayed high-affinity binding to human GHR (Kd 2.56 ± 0.61 nM; *n* = 11) and lower affinity to rat GHR (Kd 19.1 ± 4.9 nM; *n* = 6), with no detectable binding to mouse or cynomolgus monkey GHR (*n* = 4 and *n* = 2, respectively) (50). These SPR data demonstrate marked species selectivity and document a ∼8-fold weaker affinity for rat vs human receptors. GHR antagonism was quantified in primary hepatocytes (human, rat, dog, monkey) by suppression of GH-stimulated STAT5 phosphorylation (AlphaLISA) (50). For each species, an effective 80% concentration (EC_80_) of the species-specific recombinant GH was first determined; compounds were then tested in the presence of this EC_80_ GH. Across 9 replicates per condition, ALXN2420 and pegvisomant both inhibited phosphorylated STAT5 in a concentration-dependent manner. These results indicate that ALXN2420 is a functional competitive human GHR antagonist and is biologically active in rat and dog hepatocytes, exhibiting species-specific differences that partially correspond to the binding profile as determined by SPR (Biacore^™^; Cytiva, Marlborough, MA) (50, 51).

#### In vivo data

Efficacy was evaluated in healthy rats and dogs using SC ALXN2420 injections with serial serum IGF-I measurements (50). In rats, a single SC dose of ALXN2420 produced a clear, dose-dependent decrease in IGF-I at 24 hours (50). With once daily (QD) dosing, 30 mg/kg, yielded a −35% ± 5% decline of IGF-I vs baseline at 24 hours; whereas twice-daily (BID) dosing, 30 mg/kg, achieved −39% ± 8%. At the low-dose end, BID 1 mg/kg showed a modest but significant IGF-I reduction (of −7% ± 7%; *P* = .0382). By 48 hours post dose, IGF-I returned to (or slightly above) baseline across doses, indicating reversibility. In a 4-day QD injection protocol (30 mg/kg), IGF-I was lowest by 24 hours (−32% ± 8%, *P* = .0006) and remained suppressed through the dosing window and 24 hours after the last injection before rising back to baseline. Daily pegvisomant (100 mg/kg) produced a similar magnitude of IGF-I decline at the end of treatment (−26% ± 8%) (50).

In dogs, after a single SC dose of 1 and 10 mg/kg ALXN2420, IGF-I was reduced at 24 hours (−15% ± 1.1%, *P* = .0035; −34% ± 8.7%, *P* = .0406), whereas 0.1 mg/kg was ineffective (50). With QD 1 mg/kg for 5 days, suppression was evident after the first injection and maintained through day 5 (mean −21% ± 6% at 24 hours after the last dose), with levels remaining below baseline for ≥ 3 days off treatment.

Following a single SC administration, ALXN2420 reached time to maximum concentration at ∼1 hour in rats and after 2 to 4 hours in dogs (50). The terminal half-life (*t*½) was 5.19 hours in rats (1 mg/kg) and 13.21 hour in dogs (1 mg/kg), with dose-proportional increase in maximum concentration (*C*_max_) in dogs (0.1-10 mg/kg). The longer calculated half-life in dogs is concordant with the more prolonged IGF-I suppression observed relative to rats.

In addition to monotherapeutic studies, rats received continuous SC octreotide infusion (20 or 40 µg/kg/day for 4 days) with a single ALXN2420 injection on day 3 (0.3-30 mg/kg) (50). Octreotide alone reduced IGF-I by ∼10% at either dose (−10% ± 9% and −9% ± 5%); ALXN2420 alone produced dose-dependent reductions (−7% ± 8% to −29% ± 5% across 1-30 mg/kg). Combined therapy was additive; for example, 3 mg/kg ALXN2420 yielded −13% alone and 20 µg/kg/day octreotide yielded −10% alone, whereas the combination achieved an IGF-I reduction of −23%. Notably, 3 mg/kg ALXN2420 + octreotide matched the maximal reduction seen with 30 mg/kg ALXN2420 monotherapy (*P* = .9974), implying an ∼10-fold left shift in the ALXN2420 dose requirement at maximal effect under SRL cotreatment (50).

No specific adverse clinical signs or laboratory toxicities were detailed in these efficacy studies in animals.

#### Clinical trials

The phase 1 ALXN2420 study was presented at the Endocrine Society Meeting in 2024 under the compound's former name, AZP-3813 (52). In this randomized, double-blind, placebo-controlled, single ascending dose (SAD) and multiple ascending dose (MAD) trial, safety, tolerability, PK, and PD after SC injection of ALXN2420 were assessed. In the SAD, 1 cohort received a single SC injection of 3 mg (*n* = 5; ALXN2420:placebo = 3:2); 6 further cohorts received 10, 20, 40, 60, 90, or 120 mg (each *n* = 8; ALXN2420:placebo = 6:2). Dose-related IGF-I reduction was observed with doses ≥ 10 mg with more prolonged suppression (up to ∼72 hours) at higher single doses. In the MAD study, 3 cohorts received 10, 20, or 40 mg or placebo once daily SC for 14 consecutive days (each *n* = 8; ALXN2420:placebo = 6:2). ALXN2420 showed dose-proportional increases in *C*_max_ and AUC, with an estimated *t*½ of 18 to 22 hours. Pharmacodynamic studies showed a gradual, sustained, dose-related IGF-I lowering at 20 and 40 mg/day, with larger effects observed after 2 weeks of daily ALXN2420 injection than after a single dose, demonstrating a cumulative effect of repeated dosing. Mean placebo-adjusted percent change from baseline at day 14 was −19% (20 mg) and −44% (40 mg), respectively (52). Across the SAD and MAD cohorts, ALXN2420 was described as generally well tolerated and no safety concerns were reported (52).

A phase 2, multinational, randomized, double-blind, placebo-controlled, parallel-group, dose-range–finding study with an OLE (NCT07037420) aims to include adult patients with active acromegaly on stable, maximally tolerated, long-acting SRL therapy (octreotide or lanreotide) for ≥ 6 months who remain partial responders, defined as a > 20% IGF-I reduction during SRL therapy (53). The primary outcome of the 15-week blinded period (ALXN2420 vs placebo) is the proportion of participants achieving IGF-I ≤ 1.3 × ULN at week 15.

### MAR002

**Figure 2:**
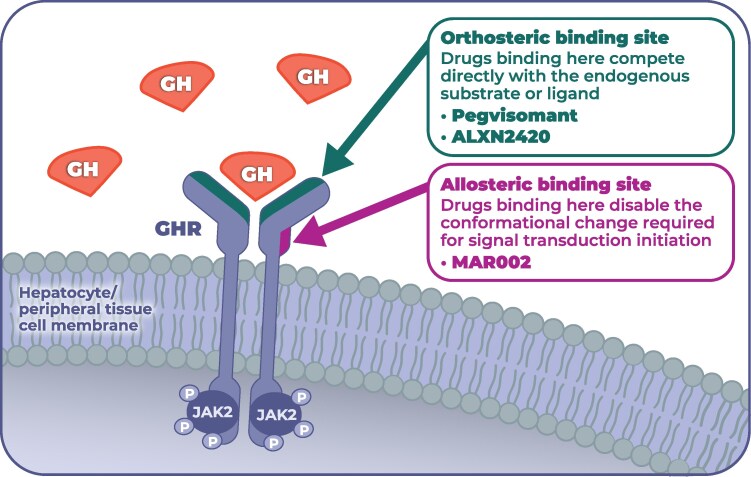
Orthosteric vs allosteric inhibition of the GH receptor. Schematic of a dimeric GHR in the hepatocyte/peripheral tissue membrane. Orthosteric antagonists (pegvisomant; ALXN2420) bind the GH binding site and compete with endogenous GH, preventing subsequent receptor activation. The allosteric antagonist MAR002 engages a distinct epitope, prevents the rotation-like conformational change normally induced by GH binding to the receptor-dimer, and therefore blocks signaling independent of GH binding. Both the orthosteric and allosteric mechanisms inhibit downstream JAK2-coupled signaling. Schematic only; not to scale. Adapted from a figure created with BioRender. Abbreviations: GH, growth hormone; GHR, GH receptor; JAK2, Janus kinase 2.

MAR002 is a half-life-extended, allosteric, human monoclonal antibody against the GHR in development for the treatment of acromegaly by Marea Therapeutics (San Francisco, USA) (54, 55). It antagonizes GHR signaling without preventing GH binding, indicating a GH occupancy–independent, allosteric mechanism distinct from orthosteric antagonism (see Fig. 2) (54, 55). In vitro, MAR002 inhibits GH-induced GHR signaling with a half maximal inhibitory concentration (IC_50_) of 1 nM, whereas pegvisomant shows an IC_50_ of 122 nM in the same assay (54). MAR002 maintains GHR signaling suppression when GH is prebound to GHR and at supraphysiologic GH concentrations, under which pegvisomant does not suppress signaling. Epitope mapping demonstrates that MAR002 does not bind known GH binding sites on GHR and can simultaneously occupy GHR with GH (54). In cynomolgus monkeys, equimolar single doses of MAR002 and pegvisomant achieve similar maximal IGF-I lowering of approximately 80% (54). The duration of ≥ 50% IGF-I suppression is prolonged with MAR002 (about 36 days) compared with pegvisomant (about 15 days) (54, 55). Half-life–extension features and known interspecies PK scaling support a prediction of longer IGF-I lowering in humans than in monkeys (54). Consequently, MAR002 is being developed for infrequent SC administration.

In a first-in-human, randomized, blinded, parallel-group, placebo-controlled, single ascending dose study with MAR002 (MAR-201, NCT07195175) in healthy men (56), the primary objective is to assess safety and tolerability after SC dosing. Secondary objectives included characterization of PK. Exploratory objectives included effects on serum IGF-I and assessment of antidrug antibodies (56). Marea Therapeutics proposes a potential combination of MAR002 with SRLs for patients with acromegaly (55). Compared with pegvisomant, MAR002 aims to reduce injection burden while maintaining robust IGF-I–lowering effects through an allosteric mechanism and extended duration of action (54, 55).

## Investigational agents discontinued after clinical testing

Other investigational agents for acromegaly advanced to clinical testing in recent years (51), but their development was subsequently discontinued. Two products were antisense oligonucleotides that target GHR messenger RNA and promote receptor degradation, leading to IGF-I reduction: ATL1103 (atesidorsen, Percheron Therapeutics, discontinued 2025) (57) and IONIS GHR LRx (cimdelirsen, Ionis Pharmaceuticals, discontinued 2023) (58). An oral small molecule with selective agonist activity at SST2 (ONO 5788, Ono Pharmaceutical) was discontinued in 2019 (59).

## Summary

Recently approved options for treating acromegaly are oral paltusotine (Palsonify^™^), approved in the United States, and an octreotide SC depot (CAM2029, Oczyesa^®^), approved in Europe, which broaden SRL therapy through oral and ready-to-use SC administration, respectively.

Ongoing development focuses on octreotide formulations that aim to reduce injection frequency, with Debio 4126 intended for IM administration every 3 months. Furthermore, GHR antagonism with ALXN2420, a bicyclic peptide, is being tested as an add-on therapy to SRLs, while MAR002, an allosteric monoclonal antibody, is in initial in-human evaluation.

## Future directions

Looking ahead, we believe that optimizing acromegaly outcomes will require an integrated strategy that explicitly addresses both hepatic and extrahepatic acromegaly. Adenoma-targeted SRL therapy remains essential for suppressing GH secretion and controlling tumor biology. However, new SRL formulations should deliver a more uniform PD effect across the dosing interval to mitigate intracycle variations in IGF-I and symptom burden documented with currently available SRLs (60, 61). In parallel, GHR-targeted pharmacotherapy can blunt residual peripheral GH action, thereby complementing SRL-mediated source control to achieve comprehensive regulation of both the hepatic (IGF-I–mediated) and extrahepatic manifestations of disease. We anticipate that such combination therapy will normalize mortality—largely determined by biochemical control—and meaningfully reduce morbidity and functional impairment, domains inadequately captured by suppressing IGF-I alone.

## Data Availability

Data sharing is not applicable to this article as no data sets were generated or analyzed during the present study.

## Abbreviations

AcroQoL: Acromegaly Quality of Life Questionnaire
AUC: area under the curve
BID: twice a day
CI: confidence interval
*C*_max_: maximum concentration
EC_80_: effective 80% concentration
EMA: European Medicines Agency
FDA: US Food and Drug Administration
GH: growth hormone
GHR: growth hormone receptor
IC_50_: half maximal inhibitory concentration
IGF-1: insulin-like growth factor 1
IM: intramuscular
JAK2: Janus kinase 2
LAR: long-acting release
LC–MS/MS: liquid chromatography-tandem mass spectrometry
MAD: multiple ascending dose
MRI: magnetic resonance imaging
OLE: open-label extension
PD: pharmacodynamics
PK: pharmacokinetics
Q12W: every 12 weeks
QD: once daily
SAD: single ascending dose
SC: subcutaneous
SPR: surface plasmon resonance
SRL: somatostatin receptor ligand
SST2: somatostatin receptor 2
SST5: somatostatin receptor 5
STAT5: signal transducer and activator of transcription 5
t½: terminal half-life
ULN: upper limit of normal
